# Case of Artificial Nose and Palate

**Published:** 1850-04

**Authors:** W. E. Ide

**Affiliations:** Columbus, Ohio.


### ARTICLE III.

### *Case of Artificial Nose and Palate.* By W. E. Ide, M. D., Columbus, Ohio.

Mr. H. C., aet. about fifty, applied at my office, about six months since, for an artificial nose. On examination, I found the following to be his condition:

The whole of the nose, including the entire nasal bones, were wanting, and in its place were two openings, or holes; the upper, of an oval shape, about an inch in diameter, in a vertical, and three-fourths of an inch in a horizontal direction; and the lower, round, and about one-half an inch in diameter. The lower opening was on a line with the palatine plate of the maxillary bone, and the upper occupied the place of the nasal bones. The vomer, and turbinated portions of the ethmoid bones were entirely wanting, together with the septum palati. The front portions of the maxillary bones, at their union on the median line, had been removed, leaving an aperture about one-third of an inch in diameter, from the mouth to the anterior nares, and about the centre of the hard palate was another opening, about five-eighths of an inch in diameter.

The teeth of the upper jaw had all been removed, and the velum palati had been partly destroyed, and adhered, to some extent, to the posterior wall of the fauces.

The disease which had resulted so disastrously, also destroyed the right arm, but had ceased nearly ten years since. The general health was now pretty good; but articulation was, of course, very much impaired, and mastication was attended by many annoying difficulties. In the first place, the openings into the nares destroyed the power of suction in taking of fluids, and when taken, they were liable to rush through them. In mastication, the food was often forced through the same openings, and had to be removed, with much difficulty, through the facial apertures. The lachrymal and mucous secretions were almost constantly *dripping* through the lower facial opening, to the great annoyance of the friends, as well as to the patient himself.

After a careful examination, I inquired if he would submit to some arrangement for the artificial closure of these bucco-nasal openings? He replied, that he would consider it of the greatest importance, could it be done; but that he had applied to so many dentists for that purpose, who had uniformly declared it to be impossible, that he had long since given up the idea, and now contented himself with the prospect of an artificial nose only. Having informed him that I thought it quite possible, he at once consented that I should make a trial.

My first plan would have been to make an entire artificial palate, attaching to it a set of teeth; but the orbicularis oris had been so involved in the disease, as in cicatrizing, to contract to that degree that an impression of the *whole* upper jaw could not possibly be taken. I then concluded to proceed as follows:

An impression of the *central portion* of the jaw was taken, including the openings and their vicinity. After making the dies, a plate was struck up that covered this part of the mouth, and extended forward to that part where the alveolar arch had been removed. To this plate wax was then applied to represent the last substance of the interrupted arch, and from this, another impression, dies and plate, were made. This plate was then soldered to the former, forming a hollow box, and then, to the upper surface of the whole was applied *soft* wax, and the fixture put in its place in the mouth, to work the position of the holes in the roof. On the place so marked, were soldered two cones, about three-fourths of an inch in length, and in shape and diameter, at the base, representing the openings which they were designed to close. To the apex of the posterior cone was soldered a strong wire, the upper end terminating in a small ring or loop. This wire was of such length, and so bent, as when the fixture was *in situ*, to reach to the upper edge of the upper *facial* or *nose cavity.*

The next step was, to take an impression, in plaster, of the central portion of the face, including the nasal cavities, and extending upward to the frontal bone, and downward to the mouth. From this, a cast and die were made. To that portion representing the upper nasal cavity a plate was fitted that should cover said cavity, and extend from one-half to one-third of an inch beyond, and around it in every direction. The centre, corresponding in shape and size with this cavity, was then cut out, and into this was soldered a flange, half an inch deep. This flange was set into, and just filled the facio-nasal cavity, and the plate rested on the facial surface. To the upper part of this collar, or double flange, a small hook was attached to receive the ring or loop of the wire, that was attached to, and supported the palatine plate; and to the lower and front surface was attached a socket, to receive and support the artificial nose.

The artificial nose was then made in the following manner: A plaster cast was taken, from the nose of a person having one of about the desired shape and size. The inner surface of this being oiled, plaster was poured in, till it was filled. When the plaster had *set*, the mould was broken away from it, leaving a perfect representation of the nose and nostrils. This was then carved down on the facial surface, till it fitted the cast that had previously been taken of the patient's face, and to this cast it was attached by wax. From this, two dies were cast in zinc; one representing the nose, and the other the nostrils. A silver plate was then struck up, representing the former, and another representing the latter, and then the two soldered together; thus forming the entire nose,—the base slightly flanging out upon, and perfectly fitting the face. A wire, about an inch and a half long, was soldered at one end to the bridge of the nose, on the inner side, and the other end bent to a right angle with itself, to be received in the socket of the *double flange,* before described. In the upper part of the nose was made a small hole to receive a pin, which was attached to the centre of the bone of the spectacles.

The fixture being complete, the patient was able, with his one hand, to apply the palatine plate, to hook its supporting wire to the double flange, to adjust the nose, or to remove the same, without difficulty.

To give color to the artificial nose, it was partially covered with enamel, and painted. To prevent the secretions of the nares from passing out through the lower facial opening, a hollow *metallic stopper* was made, which perfectly answered the purpose, and was concealed by the nose.

The voice of the patient was almost entirely restored; being only slightly affected by the adhesions of the velum palati, and all the difficulties attending mastication were removed.

### ARTICLE IV.

*Neuralgia* VS. *Tooth-ache.* By S. M. SHEPHERD, D. D. S. *of Petersburg, Va.*

